# Quantitative assessment of physical activity in pregnant women with sonographic short cervix and the risk for preterm delivery: A prospective pilot study

**DOI:** 10.1371/journal.pone.0198949

**Published:** 2018-06-11

**Authors:** Roni Zemet, Eyal Schiff, Zipora Manovitch, Tal Cahan, Rakefet Yoeli-Ullman, Benny Brandt, Israel Hendler, Lilia Dorfman-Margolis, Yoav Yinon, Eyal Sivan, Shali Mazaki-Tovi

**Affiliations:** 1 Department of Obstetrics and Gynecology, Sheba Medical Center, Tel-Hashomer, Israel; 2 Sackler School of Medicine, Tel Aviv University, Tel Aviv, Israel; University of Illinois at Urbana-Champaign, UNITED STATES

## Abstract

**Objective:**

Bed rest or activity restriction is a common obstetrical practice, despite a paucity of data to support its efficacy. The aim of this study was to determine whether physical activity, as assessed by a smart band activity tracker, is associated with preterm birth in pregnant women at high risk for preterm delivery.

**Methods:**

This was a pilot prospective cohort study including pregnant women at high risk for preterm delivery between 24 and 32 weeks-of-gestation. Physical activity level was assessed by smart band activity. Patients with sonographic short cervical length (≤ 20 mm) were asked to wear the smart band activity tracker continuously for at least one week, including one weekend. Both physicians and patients were blinded to the data stored in the smart band activity tracker. No specific recommendations were given to participants as to the level or intensity of physical activity. The primary outcome was the rate of preterm birth (< 37 weeks-of-gestation). Secondary outcomes included the rate of delivery before 34 weeks of gestation and neonatal outcome. Parametric and nonparametric statistics were used for analysis.

**Results:**

Study population included 49 pregnant women: 37 women (75.7%) delivered preterm and 12 (24.5%) delivered at or after 37 weeks-of-gestation. The median steps per day was significantly lower in patients who delivered preterm (3576, IQR: 2478–4775 vs. 4554, IQR: 3632–6337, p = 0.02). Regression analysis revealed that the median number of steps per day was independently inversely associated with preterm birth, after adjustment for maternal age, body mass index, gestational age at recruitment, cervical length, cervical dilatation and plurality.

**Conclusion:**

This pilot study represents the first quantitative assessment of the association between physical activity and preterm birth. The results of this pilot study do not support the efficacy of decreased physical activity in the prevention of preterm birth in patients with sonographic short cervical length.

## Introduction

Bed rest or activity restriction in hospital or at home is a very common obstetrical practice. Approximately 95% of obstetricians report recommending bed rest for various indications, and 18% of pregnant women in the United States will be placed on bed rest at some point during their pregnancies [1]. Bed rest has been prescribed for women with several obstetric complications, including threatened abortion, preterm labor, preterm premature rupture of membranes, fetal growth restriction, gestational hypertension, preeclampsia and multiple gestations, with the hope of prolonging pregnancy [2]. The rationale for this treatment is based on the hypothesis that hard work and strenuous physical activity during pregnancy could be associated with preterm birth [3], and on the notion that bed rest could reduce uterine activity [2].

Despite the common use of bed rest in obstetrics, only a limited number of randomized trials have been performed to evaluate its efficacy. A Cochrane review on bed rest for prevention of miscarriage analyzed two studies including 84 women [4]. There was no statistically significant difference in the risk of miscarriage in the bed rest group versus the control group (RR 1.54, CI 0.92–2.58). Insofar as the prevention of preterm birth, current evidence does not support or refute the use of bed rest. A recent Cochrane review on bed rest for prevention of preterm birth included data of 1266 women comparing bed rest with placebo or no intervention [5]. Preterm birth before 37 weeks was similar among groups (RR 0.92, CI 0.62–1.37). Furthermore, there is no evidence that bed rest decreases the prevalence of preterm delivery in women with short cervix [6]. Routine bed rest as a course of treatment in multiple gestation pregnancies lacks evidence support. Cochrane review from 2010 on bed rest for multiple pregnancy included seven trials with 713 women [7]. Bed rest did not reduce the risk of preterm birth or perinatal morbidity.

A notable limitation of prior studies is the absence of quantitative and continuous record of the patients’ level of activity. Furthermore, assessment of patient’s adherence to bed rest is very challenging without quantitative assessment of physical activity [8]. One way to overcome this problem is the use of a portable electronic device that counts every step a person takes such as pedometers and a smart-band activity tracker.

To the best of our knowledge, quantitative assessment of physical activity in pregnant women at high risk for preterm birth, one of the most common indications for bed rest, has not been reported. Thus, the aim of this study was to evaluate whether physical activity, as assessed by the smart-band activity tracker, is associated with preterm delivery in pregnant women at high risk for preterm birth.

## Materials and methods

### Subjects

This pilot study was a prospective cohort study including women at high risk for preterm delivery between 24 and 32 weeks of gestation. All women were recruited immediately upon diagnosis of short cervix or cervical dilatation and all were hospitalized for assessment in the high risk unit at a single tertiary care center. Inclusion criteria included: 1. Sonographic cervical length ≤20 mm; 2. Viable pregnancy; 3. Spontaneous preterm birth. Exclusion criteria included: 1. Preterm labor; 2. Clinical signs or symptoms of chorioamnionitis; 3. Preterm premature rupture of membranes (PPROM); 4. Medically indicated preterm birth; 5. Pregnancies complicated with congenital anomalies or chromosomal abnormalities; 6. fetal death. Three patients have declined participation.

### Clinical definitions

Gestational age was determined by an ultrasound examination in the first trimester. Preterm labor was defined as the presence of regular uterine contractions occurring at a frequency of at least two every 10 minutes, associated with cervical changes that required hospitalization before 37 weeks of gestation. Preterm birth was defined as birth before 37 weeks of gestation. Clinical chorioamnionitis was diagnosed by the presence of maternal fever (temperature > 37.8°C) accompanied by two or more of the following criteria: 1) uterine tenderness; 2) malodorous vaginal discharge; 3) fetal tachycardia (heart rate > 160 beats/min); 4) maternal tachycardia (heart rate>100 beats/min); and 5) maternal leukocytosis (leukocyte count > 15,000 cells/mm^3^). Maternal body mass index (BMI) was calculated upon enrollment according to the following formula: weight (Kg)/height (m)^2^. Birth weight was obtained immediately after the delivery using a standard electrical scale.

Neonatal secondary outcomes included the following: respiratory distress syndrome (RDS), transient tachypnea of the newborn (TTN), need for respiratory support (continuous positive airway pressure [CPAP], or mechanical ventilation oxygen supplementation), admission to NICU or special care unit, hypoglycemia, jaundice defined as hyperbilirubinemia requiring treatment, sepsis confirmed by positive blood cultures, suspected sepsis requiring sepsis work up, necrotizing enterocolitis (NEC), intraventricular hemorrhage (IVH) and a composite neonatal morbidity outcome which included RDS, TTN, sepsis or a need for respiratory support. The respiratory distress syndrome was defined as the presence of clinical signs of respiratory distress (tachypnea, retractions, flaring, grunting, or cyanosis), with a requirement for supplemental oxygen with a fraction of inspired oxygen of more than 0.21 and a chest radiograph showing hypoaeration and reticulogranular infiltrates. Transient tachypnea of the newborn was diagnosed when tachypnea occurred in the absence of chest radiography or with a radiograph that was normal or showed signs of increased perihilar interstitial markings and resolved within 72 hours. Hypoglycemia was defined as a glucose level of less than 40 mg per deciliter at any time.

### Intervention

Physical activity level was assessed by continuous use of the smart-band activity tracker (Polar Loop Activity Band; Polar Electro, Kempele, Finland). The smart-band activity tracker was used according to the manufacture instructions. Women were asked to wear the smart band activity tracker continuously (including upon exposure to water), on their wrist, for at least one week including one weekend, and until 3 weeks from recruitment or delivery, whichever came first. A designated email account was created for each participant by the research team and the smart-band activity tracker was synchronized with this email account. The research team did not draw the data from the email accounts until the end of the study. Thus, both physicians and patients were blinded to the data stored in the smart-band activity tracker. No specific recommendations were given to participants as to the level or intensity of physical activity they should adhere to, however, all patients were instructed to remove from work upon discharge from hospitalization. All patients with short cervix were treated with antenatal corticosteroids (2 doses of 12 mg of betamethasone Intramuscularly 24 hours apart), vaginal progesterone (200 mg of micronized progesterone) and tocolysis according to standardizes protocol. Compliance was determined by analyzing the data retrieved from the activity tracker band.

The primary outcome was rate of preterm birth (delivery before 37 weeks of gestation). Secondary outcomes included the rate of delivery before 34 weeks of gestation and neonatal outcome.

### Validation of smart band activity tracker

Agreement between the smart-band activity tracker and the validated pedometer [9, 10] (Omron HJ-720ITC, Omron Healthcare Inc; Bannockburn, Illinois, USA) was determined in 20 pregnant women not included in the study group. Pregnant women were asked to wear both the smart-band activity tracker and the pedometers for at least 24 hours. Agreement between the smart-band activity tracker and the validated pedometer was assessed in several ways. Absolute step-count measurements were compared using the Wilcoxon Signed-Rank Test. The relative agreement between pedometer-derived step-counts and smart-band activity tracker examined by determination of the Spearman rank correlation coefficient. The classification of participants according to whether or not they recorded a daily mean of at least 3,800 steps/day (median number of steps in pooled analysis) was compared between pedometer and smart-band activity tracker by calculating Cohen’s kappa over 2 × 2 contingency tables.

### Statistical analysis

Normality of the data was tested using the Shapiro-Wilk or Kolmogorov-Smirnov tests. Data are presented as median and inter-quartile range (IQR). Comparison between unrelated variables was conducted with Student's t-test or Mann–Whitney U test, as appropriate. The chi-square and Fisher's exact tests were used for comparison between categorical variables. Correlation between variables was conducted using either Pearson or Spearman’s rank correlation as appropriate. Logistic regression analysis was used to determine which factors were significantly and independently correlated with preterm delivery. Significance was accepted at p < 0.05. Statistical analyses were conducted using the IBM Statistical Package for the Social Sciences (IBM SPSS v.19; IBM Corporation Inc, Armonk, NY, USA).

The study protocol was approved by the Institutional Review Board at the Sheba Medical Center (no. 1197-14-SMC), and all patients provided a written informed consent.

This trial is registered with ClinicalTrials.gov, number NCT02343848.

## Results

Study population included 49 pregnant women. Women were recruited at a single tertiary care center between 2014 and 2016. In pooled analysis, the rate of preterm birth was high (75.5%). The rate of delivery before 34 and 32 weeks of gestation was 40.8% and 30.6%, respectively. The median gestational age at delivery was 34.5 weeks (IQR 31.2–36.8). The study population included 23 pregnant women with singleton and 26 with multifetal gestation. The rate of preterm birth was relatively high as well among those with singleton pregnancies in the group (65.2%).

Participants wore the smart-band activity tracker for a median of 15 days (IQR 9.5–21). The median sonographic cervical length was 9 mm (IQR 6.5–12.5 mm). As expected, the median number of steps in pooled analysis was relatively low in comparison to pregnant women with no increased risk for preterm labor as reported in previous published studies [11, 12]: 3871 (IQR: 2925–4829). There was no significant difference in the median number of steps per day between women with singleton (3801, IQR 3024–4857) and multifetal gestation (3920, IQR 2757–4878, p = 0.98).

### Physical activity and preterm birth

**Fig 1:**
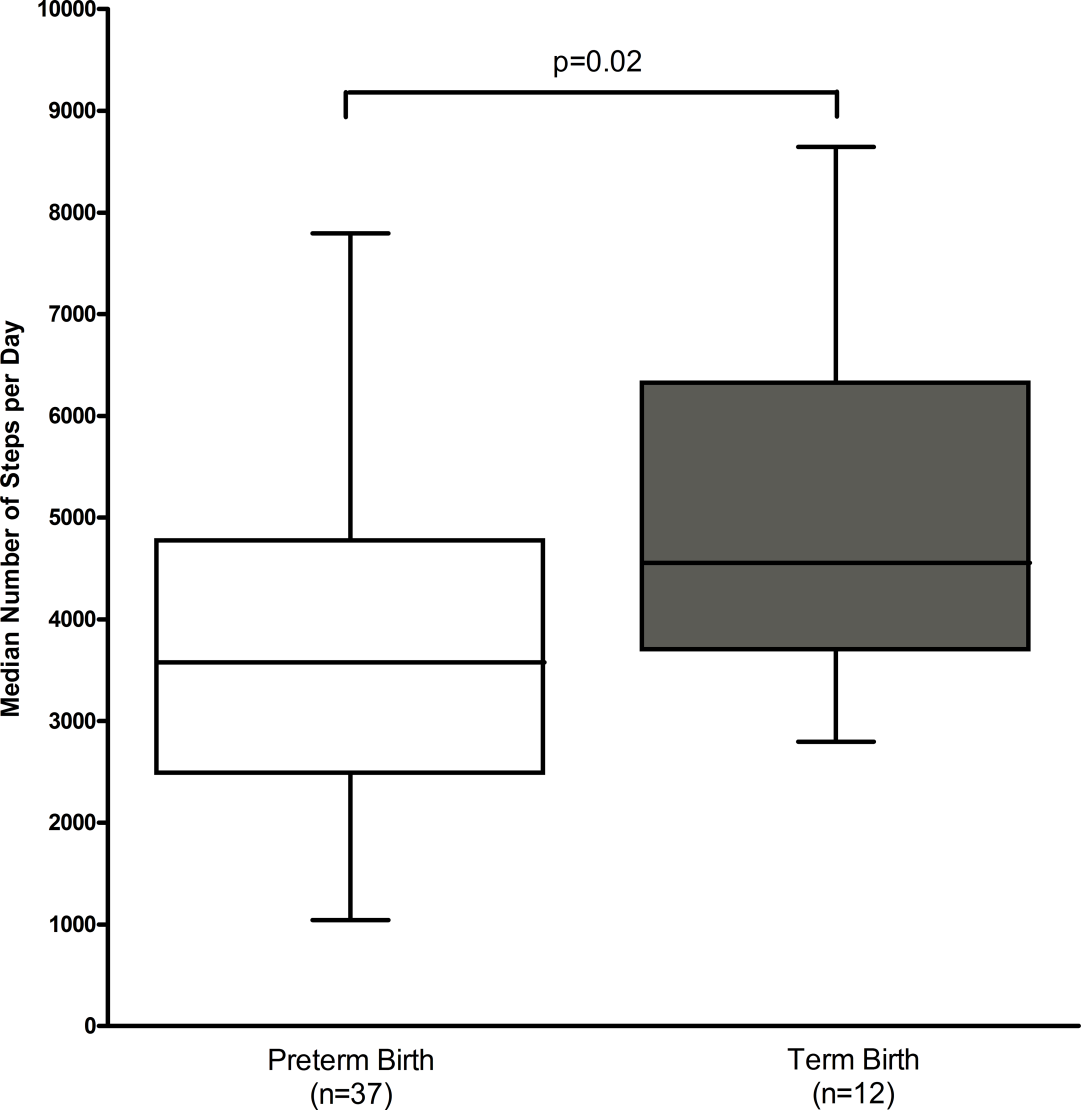
Comparison of physical activity in pregnant women delivered before and after 37 weeks of gestation.

Table 1 displays the demographic and clinical characteristics of pregnant women delivered before and after 37 weeks of gestation. The median steps per day was significantly lower in women who delivered preterm (3576, IQR: 2478–4775 vs. 4554, IQR: 3632–6337, p = 0.02, Fig 1). Other demographic and clinical characteristics, including cervical length and gestational age at recruitment, and the rate of multiple gestation, did not differ significantly between the two groups.

**Table 1:** Demographic and clinical characteristics of study group as a function of gestational age at delivery: term vs. preterm.

Characteristics	Delivery before 37 weeks (n = 37)	Delivery at or after 37 weeks (n = 12)	p value
**Gestational age at recruitment (weeks)**	26.0 (24.5–29.1)	26.5 (24.8–30.3)	0.53
**Gestational age at delivery (weeks)**	33.2 (30.5–35.1)	38.1 (37.5–39.1)	0.001
**Maternal age (years)**	30.5 (27.5–33.5)	33.0 (29.5–38.2)	0.24
**Pregestational BMI (kg/m**^**2**^**)**	21.4 (19.1–25.5)	20.7 (18.7–24.9)	0.91
**BMI at recruitment (kg/m**^**2**^**)**	25.1 (22.2–29.4)	25.2 (23.7–28.4)	0.85
**Gravidity**	2.0 (1–3.5)	1 (1–2.7)	0.53
**Parity**	0 (0–1.0)	0 (0–1.5)	0.67
**Nulliparous, n (%)**	17 (45.9)	7 (58.3)	0.34
**Sonographic cervical length (mm)**	9 (5–12)	12 (8.1–14)	0.07
**Cervical dilatation (cm)**	0 (0–1)	0 (0–0)	0.2
**Days of activity measurement**	16 (9.5–23)	13 (9.5–15.5)	0.14
**Singleton, n (%)**	15 (40.5)	8 (66.7)	0.18
**History of preterm birth, n (%)**	4 (10.8)	2 (16.6)	0.46
**Cerclage, n (%)**	3 (8.1)	2 (16.6)	0.58
**Steps per day**	3576 (2478–4775)	4554 (3632–6337)	0.02

Data are presented as median and interquartile range (IQR). BMI—Body Mass Index

The rate of RDS (27% vs. 0%, p = 0.04), admission to NICU or special care unit (75.7% vs. 16.5%, p = 0.001), and composite neonatal morbidity (29% vs. 0%, p = 0.001) was significantly higher in the preterm group. Infants of pregnant women delivered preterm and at term did not differ significantly in the rate of NEC (2% vs. 0%, p = 0.76), IVH (5% vs. 0%, p = 0.56) or sepsis (11% vs. 0%, p = 0.3).

The association between preterm birth, physical activity and possible confounding factors was further studied by regression analysis. Median number of steps per day (p = 0.02) was independently associated with preterm labor after adjustment for maternal age, maternal BMI, gestational age at recruitment, cervical length, cervical dilatation and plurality (Table 2).

**Table 2:** Regression analysis of factors associated with preterm labor.

Factor	Exp (B)	95% CI	p value
**Median steps per day**	1.001	1.000–1.001	0.02
**Maternal age**	1.071	0.919–1.249	0.38
**Gestational age at recruitment**	1.125	0.801–1.581	0.49
**Pregestational BMI**	1.059	0.832–1.335	0.64
**Cervical length**	1.011	0.765–1.335	0.94
**Cervical dilatation**	0.142	0.01–2.05	0.15
**Plurality**	0.396	0.069–2.272	0.29

BMI—Body Mass Index

### Physical activity and delivery before vs. after 34 weeks of gestation

20 pregnant women delivered before 34 weeks of gestation while 29 delivered at or after 34 weeks of gestation. Table 3 displays the demographic and clinical characteristics of pregnant women who delivered before and after 34 weeks of gestation. The median steps per day did not differ significantly between women who delivered before 34 weeks compared to those who delivered after 34 weeks (3530, IQR: 2876–4874 vs. 3969, IQR: 3068–4788, p = 0.72). Other demographic and clinical characteristics did not differ significantly between the two groups.

**Table 3:** Demographic and clinical characteristics of study group as a function of gestational age at delivery: before and after 34 weeks of gestation.

Characteristics	Delivery before 34 weeks (n = 20)	Delivery at or after 34 weeks (n = 29)	p value
**Gestational age at recruitment (weeks)**	26.0 (24.5–28.0)	27 (24.6–30.2)	0.28
**Gestational age at delivery (weeks)**	30.9 (30.1–31.8)	36.4 (35.1–38.0)	<0.001
**Maternal age (years)**	31.0 (27.5–35.2)	31.0 (28.5–34.0)	0.93
**Pregestational BMI (kg/m**^**2**^**)**	21.3 (19.7–25.1)	21.0 (19.0–25.5)	0.88
**BMI at recruitment (kg/m**^**2**^**)**	25.4 (23.1–29.1)	24.8 (22.2–29.4)	0.74
**Gravidity**	1.5 (1–2)	2 (1–3.5)	0.81
**Parity**	0 (0–1.0)	0 (0–1.0)	0.91
**Nulliparous, n (%)**	10 (50)	14 (48.3)	0.56
**Sonographic cervical length (mm)**	8 (5–11.5)	12 (8.0–13)	0.11
**Cervical dilatation (cm)**	0.5 (0–1)	0 (0–0.5)	0.1
**Days of activity measurement**	18.5 (12.2–25.2)	14 (9.0–17.0)	0.09
**Singleton, n (%)**	8 (40)	15 (51.7)	0.56
**History of preterm birth, n (%)**	2 (10)	4 (13.7)	1.00
**Cerclage, n (%)**	2 (10)	3 (10.3)	1.00
**Steps per day**	3530 (2876–4873)	3969 (3068–4788)	0.72

Data are presented as median and interquartile range (IQR). BMI—Body Mass Index.

The rate of RDS (before 34 weeks: 50% vs. after 34 weeks: 0%, p = 0.001), need for respiratory support (35% vs. 0%, p = 0.001), admission to NICU or special care unit (90% vs. 41%, p = 0.001), jaundice (65% vs. 20.7%, p = 0.001), and composite neonatal morbidity (55% vs. 3.4%, p = 0.001) was significantly higher in the newborns delivered before 34 weeks of gestation compared to those delivered after 34 weeks. Pregnant women delivered before and after 34 weeks of gestation did not differ significantly in the rate of NEC (5% vs. 0%, p = 0.42), IVH (10% vs. 0%, p = 0.16), hypoglycemia (30% vs. 20.7%, p = 0.34) or sepsis (15% vs. 3.4%, p = 0.18).

### Gestational age at delivery as a function of median steps per day: ≤ 25^th^ percentile vs. > 75^th^ percentile

In order to gain further insights into whether reduced physical activity would be beneficial to reduce the rate of preterm birth, we divided the study population according to the median number of steps per day into two group: below the 25^th^ vs. above the 75^th^ percentile. The median gestational age at delivery did not differ significantly between pregnant women in the 25^th^ vs. 75^th^ percentile (35.1, IQR: 31.6–36.4 vs. 35.4, IQR: 30.0–38.8, p = 0.68). Similarly, other demographic and clinical characteristics were similar between the two groups (Table 4).

**Table 4:** Demographic and clinical characteristics of study group as a function of median steps per day: ≤ 25^th^ percentile vs. > 75^th^ percentile.

	median steps per day		
Characteristics	Less than 25^th^ percentile (n = 13)	More than 75^th^ percentile (n = 12)	p value
**Gestational age at recruitment (weeks)**	26.4 (24.5–29.3)	25.5 (24.3–29.7)	0.97
**Gestational age at delivery (weeks)**	35.1 (31.6–36.4)	35.4 (30.0–38.8)	0.68
**Maternal age (years)**	29.0 (27.0–31.5)	32.0 (27.2–39.2)	0.15
**Pregestational BMI (kg/m**^**2**^**)**	20.8 (18.1–25.8)	21.4 (18.4–25.0)	1.00
**BMI at recruitment (kg/m**^**2**^**)**	23.7 (21.6–28.3)	26.5 (24.5–29.9)	0.19
**Gravidity**	2.0 (1.0–2.5)	2 (1.0–4.0)	0.53
**Parity**	0 (1.0–1.5)	0 (1.0–2.0)	0.89
**Nulliparous, n (%)**	6 (46.2)	4 (33.3)	0.80
**Sonographic cervical length (mm)**	12.0 (9.0–12.5)	10.5 (5.0–15.7)	0.89
**Cervical dilatation (cm)**	0 (0–1.0)	0 (0–1.0)	0.93
**Days of activity measurement**	17.0 (11.0–21.0)	15.0 (13.0–21.0)	0.81
**Singleton, n (%)**	7 (53.8)	6 (50.0)	1.00
**History of preterm birth, n (%)**	2 (15.3)	1 (8.3)	1.00
**Cerclage, n (%)**	2 (15.3)	1 (8.3)	1.00

Data are presented as median and interquartile range (IQR). BMI—Body Mass Index.

### Physical activity and preterm birth: Singleton vs. Twins

We conducted a sub-analysis separating women carrying twins and women carrying singleton. Regarding women with twins (n = 26), the median steps per day was lower in women who delivered preterm compared to those who delivered at term, however the difference was borderline statistically significant, probably due to the lower number of participants (3616, IQR: 2526–4371 vs. 5190, IQR: 3679–8014, p = 0.09). Regarding women with singleton (n = 23), although the median steps per day was lower in women who delivered preterm compared to those who delivered at term, the difference did not reach statistical significance (3290, IQR: 2206–4326 vs. 4375, IQR: 2854–5384, p = 0.14).

### Correlation

The median number of steps per day did not correlate significantly with maternal age (r = 0.22, p = 0.12), gestational age at recruitment (r = 0.1, p = 0.45), maternal BMI (r = 0.19, p = 0.18), sonographic cervical length (r = -0.01, p = 0.94), or cervical dilatation (r = -0.01, p = 0.93).

### Validation

#### Agreement between continuous pedometer and smart-band activity tracker measures

There was no significant difference between the overall step counts recorded by the pedometer and the smart-band activity tracker (p = 0.81). Pedometer step counts were significantly correlated with smart-band activity tracker measures (r = 0.77, p<0.001).

#### Agreement between categorized pedometer and smart-band activity tracker measures

Agreement between the pedometer and the smart band activity tracker in categorising women to <3,800 or ≥3,800 steps/day was good (kappa = 0.63, p = 0.02, 95% CI: 0.33 to 0.93).

## Discussion

Activity restriction is probably the most common intervention prescribed to pregnant women at risk for preterm birth [13], which is the leading cause of perinatal morbidity and mortality [14, 15]. In addition to the lack of demonstrable benefit, bed rest has potential harms, including increased maternal thromboembolic risk, bone demineralization, muscle atrophy, cardiovascular deconditioning, maternal weight loss, maternal psychological problems and negative economic impact [2, 8, 16–19]. One of the most dangerous adverse effects of bed rest is the risk of venous thromboembolism [20]. One study found a significantly higher incidence of thrombosis in pregnant women placed on bed rest compared with no bed rest (RR of 19, CI 5–80) [21]. Trabecular bone loss (as determined by dual X-ray absorptiometry) in pregnant women on bed rest was compared with ambulatory pregnant women. Women on bed rest had an adjusted mean loss of 4.6% compared with 1.5% in the ambulatory women [22]. Bed rest may have a considerable psychological, familial, societal and financial effects on the pregnant woman and her family [19]. Common psychosocial effects include depressive symptoms, such as anxiety, hostility, and dysphoria [23]. The financial burden from loss of family income and threatened unemployment causes anxiety, and it may also increase healthcare costs [1].

Despite lack of evidence for any benefit and despite known harms, bed rest continues to have wide use in obstetrics [13]. A survey of maternal–fetal medicine specialists conducted in 2009 found that 71% would recommend bed rest for cervical dilation and arrested preterm labor and 87% would recommend bed rest for premature rupture of membranes, despite the fact that 72% and 56% felt there was minimal or no benefit to bed rest in the setting of preterm labor or PPROM, respectively [24].

A prominent limitation of prior studies designed to assess the association between bed rest and preterm birth, is the lack of a quantitative and continuous tool to determine and quantify physical activity. Moreover, assessment of patient’s adherence to bed rest is practically impossible without quantitative assessment of physical activity [8]. A pedometer and smart-band activity tracker provide a reliable estimation of physical activity. The pedometer and self-report exercise diary results correlated significantly, and the method was well accepted by pregnant women [25, 26]. Previous studies in which a pedometer was used during pregnancy included obese pregnant women [11, 12, 27–29], women at high risk for gestational diabetes mellitus [12, 30], women diagnosed with gestational diabetes mellitus [12], as well as healthy women [11].

To the best of our knowledge, this study represents the first quantitative assessment of the association between physical activity and preterm birth, using a smart-band activity tracker. The results of this pilot study indicate that the median steps per day was significantly lower in women who delivered preterm, compared to women who delivered at term. Furthermore, median number of steps per day was independently associated with preterm birth after adjustment for confounders. As expected, neonatal outcome was significantly worse in the preterm birth group. Thus, not only did the activity restriction did not result in elongation of pregnancy, but it was associated with increased risk of preterm birth and adverse neonatal outcome. Consistent with this finding, reduced physical activity was not associated with lower risk of delivery before 34 weeks of gestation. Collectively, the results of this study strongly support the notion of lack of efficacy and the potential deleterious effect of restricted physical activity in the prevention of preterm birth.

While the lack of activity restriction to prevent preterm birth is not contra intuitive, the biological plausibility to account for the higher rate of preterm delivery in women with decreased physical activity is obscure. Grobman et al. [6] have proposed that activity restriction has been associated with increased stress, anxiety and depression, which have been associated with an increased risk for preterm birth [31]. Consistent with this explanation, pregnant women with sleep deprivation, often associated with restricted activity and bed rest, have a higher risk for preterm births [32]. Finally, physical training programs implementations in patients destined for activity restriction such as heart failure resulted in decreased circulatory concentrations of proinflammatory cytokines including plasma TNF-a and IL-6 [33], both implicated in preterm labor.

Several strengths and limitations of our study should be acknowledged. First and foremost, this is a pilot study aimed to lay the groundwork for a larger study. Although a pilot study can serve an important role in treatment development, it is not, and should not, be interpreted as a large-scale study. Clearly, the observational design of the present study precludes comment on causality in the association between physical activity and preterm birth. Elucidation of molecular or cellular mechanisms to account for the association between reduced activity and initiation of labor was beyond the scope of this work. Additional limitation is the relatively small number of participants in the study. Nevertheless, despite the modest sample size we were able to report a statistically significant difference in the median number of steps between pregnant women who delivered before and after 37 weeks of gestation. Finally, the study population includes both singleton and twin gestation; nonetheless, the rate of multiple gestation was similar between women who deliver preterm and at term, there was no significant difference in the median number of steps per day between women with singleton and multifetal gestation, and the median number of steps per day was independently associated with preterm birth after adjustment for plurality. Among the strengths of our study is the novel implantation of a quantitative method for the determination of physical activity, the well-defined inclusion criteria for the study group, and meticulous statistical methods. Nevertheless, some caution should be exercised before unreservedly accepting the conclusions that restricted physical activity has harmful effects. In order to overcome the limitations of our study, to generalize our conclusions and to search for causality in the association between physical activity and preterm birth, a large-scale prospective multi-centered randomized control studies should be conducted.

In conclusion, to the best of our knowledge, this study represents the first quantitative assessment of the association between physical activity and preterm birth. The results of this study support the futility and potential deleterious effect of decreased physical activity (as determined by median number of steps per day) in a sub set of patients at high risk for preterm birth.

## Supporting information

S1 File Dataset. (XLS)
